# Obsession and Maladaptive Search for Raison D’être: A Condition That May Harm Psychological Wellbeing

**DOI:** 10.3389/fpsyg.2022.845834

**Published:** 2022-06-02

**Authors:** Tetsuya Akaishi

**Affiliations:** ^1^Division of General Internal Medicine, Tohoku University, Sendai, Japan; ^2^Department of Education and Support for Regional Medicine, Tohoku University Hospital, Sendai, Japan

**Keywords:** maladaptive search, obsession, raison d’être, self-oriented, psychological wellbeing

## Abstract

Being with raison d’être, or the meaning of living, usually has a positive effect on the psychological wellbeing of humans. The impact of an endeavor or desire to be with raison d’être on human wellbeing remains undetermined. This study investigated the potential impact of an obsession with raison d’être on human psychological wellbeing. A literature review revealed that only a limited number of studies have evaluated the relationship between attitudes toward raison d’être and psychological wellbeing. Some indicate that a pathological obsession with a self-oriented raison d’être, especially when the search is attempted *via* maladaptive ways, may eventually cause harm and distress to those who are the objects of obsession and the surrounding people. If obsessed people persist to preserve raison d’être in the community, they need to continuously demonstrate the advantage of their existence and differentiation from other members. As conceivable adaptive ways to search for raison d’être, people make efforts to enhance their talents, achieve certifications, be promoted, or dedicate themselves to volunteers. However, if these adaptive ways have failed, some obsessed people may change their processes to maladaptive ways, such as attacking or criticizing other members who are a threat to their satisfaction with raison d’être. Such maladaptive approaches in the community would harm both the obsessed and surrounding members. To date, the negative aspect of desiring for raison d’être has remained largely unevaluated. Research regarding the prevalence of pathologic obsession with raison d’être in the general population, its impact on human wellbeing, and treatability is warranted.

## Introduction

**FIGURE 1:**
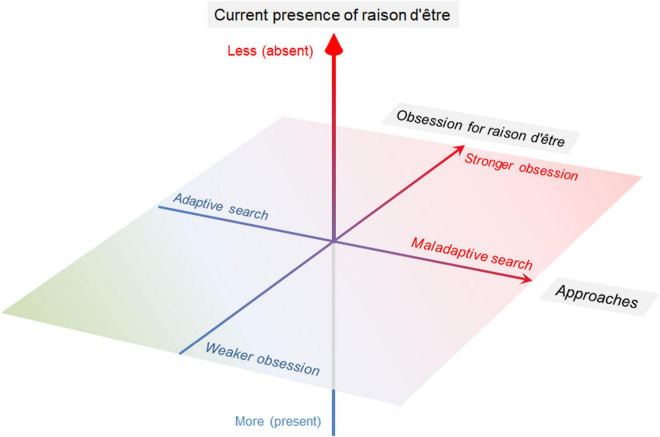
Theoretical three major components of psychological problems relating to raison d’être. The proposed three major components of the psychological problems regarding raison d’être in the present report are as follows: deficiency of raison d’être, levels of obsession with it, and maladaptive search processes. By regarding these three major components as continuous variables and not simple binomials, psychological status in each individual can be represented as a spectrum with wide variation of the psychological condition.

Humans pursue raison d’être or reasons for being in their lives (Dezutter et al., 2013). This psychological emotion to search for meaning seems inherent to almost all humans. Being with raison d’être is known to positively influence human psychological wellbeing (Zika and Chamberlain, 1992; Debats, 1996; Brassai et al., 2011). Meanwhile, due to its multidimensional character, the conceptual outlines or definitions of raison d’être remain largely undetermined (Sherman and Simonton, 2012). Furthermore, it has been proposed that being with raison d’être and the desire to search for raison d’être are distinct psychological components. The exact impact of the process of pursuing raison d’être on psychological wellbeing and the relationship between the process of doing so and mental health disorders remain largely unknown. Compared to the possession of raison d’être, attitudes toward raison d’être or searching processes for it seem to have been under-represented. It has been suggested that an adaptive search for raison d’être benefits human wellbeing (Mascaro and Rosen, 2005; deRoon-Cassini et al., 2009; Sherman et al., 2010). Meanwhile, it is reasonably expected that an abnormally strong obsession with raison d’être, especially when the search is performed *via* maladaptive behaviors, may harm psychological wellbeing, relationships with others, and attitudes toward social participation (Dezutter et al., 2013). Such obsession may even harm others in the surrounding environment. Based on these kinds of knowledge, this report hypothesized that the psychological problems regarding raison d’être are composed of several major factors, including its deficiency, levels of obsession with it, and maladaptive search for it (Figure 1). This report first describes what is currently known about the psychological impact of desiring to be with raison d’être. Then, this report further outlines the perspectives for conceivable interventions and future research in the field.

## Electronic Database Search

First, an electronic literature search of the PubMed database was performed using the following combination of keywords “(‘meaning of life’ OR ‘meaning of living’) AND obsess*”; 38 citations were identified. These citations were screened by title and abstract, and, for those that were ambiguous, the full text was reviewed. Electronic literature search was further performed with another combination of keywords “raison d’être AND (mental* OR psychological*),” which identified 22 citations. Some of the identified articles evaluated the negative impact of the absence of raison d’être on mental health status or psychological wellbeing (Isaac et al., 2014; Wand et al., 2018), but did not focus on the process of pursuing raison d’être. As the electronic search revealed a relative lack of literature in the relevant field, a narrative review by hand searches of articles on attitudes toward gaining raison d’être, including those narrative or descriptive in nature, is undertaken in the following sections.

## Adaptive Search for Raison D’être

What do we usually plan and act to make ourselves believe that we are worth being in society and equipped with a raison d’être? Meaning in life will be produced from multidimensional sources, but the most popular way is through an endeavor to establish a solid human relationship or the achievement of social positions and prestigious fames. These achievements will relieve us that we are connected to and needed from society, and they will increase the chance of survival in most cases. To succeed in these attempts, some people make enormous and desperate efforts to enhance their talents or achieve respectable certification licenses to differentiate themselves from others. Becoming famous, by appearing on mass media or utilizing social media, would be a conceivable way of gaining raison d’être so that they can believe that they are worth being in society. However, becoming famous is often accompanied by increased opportunities to be socially attacked or offended. If one cannot endure such offensive attitudes from others after becoming famous, it would be a maladaptive way for that person. The probability of succeeding in attempts to gain raison d’être increases when the motivation is other oriented and not self-oriented (Steger et al., 2008). For example, dedicating ourselves to people in need through social activities like volunteers and donations, both of which may appear to be inefficient for our prosperity or survival, is known to be effective for nourishing our raison d’être without causing conflicts with others and can be regarded as an adaptive search process (Thoits and Hewitt, 2001; Veerasamy et al., 2013; Yeung et al., 2017). However, whether self- and other-oriented motivations are incompatible emotional acts that are mutually exclusive remains uncertain. In most cases, our behavior selection seems to be based on the balance between self- and other-oriented purposes. Whether our approaches to raison d’être incline to self- or other-oriented can be objectively decided by the people surrounding us, not by ourselves.

## Maladaptive Search for Raison D’être

Ideally, it would be best for us if raison d’être could be gained and preserved through our daily lives and social activities. However, the actual world is not so simple. Establishing a positive relationship with all surrounding people and persisting with other-oriented motivations are not easy. Almost all people are vulnerable to intermittent exposure to stressful events throughout their lives, such as human conflict, bullying, harassment, demotion, dismissal, poverty, divorce, bereavement, chronic illness, or social isolation. Each of these stressful events is surely a formidable crisis against raison d’être. If someone is obsessed with raison d’être and desperate to gain it from the context of social interactions, the person must continuously suffer from the fear of being omitted from society and lose raison d’être. As no one can escape aging, the concept of ageism will be a threat to all humans for depriving our raison d’être sooner or later. Persistence to raison d’être with a maladaptive approach will inevitably result in negative feelings, such as emptiness or self-insufficiency, and may even cause envy toward others who are fulfilled with raison d’être (Steger et al., 2008; Schwartz et al., 2011). These negative emotions may cause distress to themselves and surrounding people (Dezutter et al., 2013). Using superiority to others in their skills or talents for gaining raison d’être would eventually fail to be a maladaptive way.

Another conceivable maladaptive way to gain raison d’être would be to expect approval from others. If we continuously seek approval from others in our social activities, we must endlessly continue to prove ourselves as having advantages, something special in the community, and superiority to other community members. During these attempts, we always fear the growth of our talented junior colleagues. Obsessed people are often skilled, ambitious, and workaholics. These people may be more likely to be promoted, as they tend to demand perfection in themselves (Melrose, 2011). These attitudes may be adaptive unless they harm themselves or other community members. However, once it starts to harm obsessed people or their surrounding people, it will become a maladaptive way and must be managed. If once obsessed people find it difficult to prove themselves to be superior to others in skills and talents, the alternative option left to them would be attacking or denying the existence of others, who are threats to their raison d’être and survival in the community. People who are obsessed with raison d’être and attacking others are not always mentally distressed, because they believe that they are doing the right things for their survival and even for the community. As the current targets of psychiatry are those who are mentally distressed, they are not considered targets of treatment or intervention. However, regardless of whether offenders notice that they are offending others, they are better managed psychologically or psychiatrically. To avoid further conflicts, such approaches for overcoming the obsession should be spontaneously attempted by the obsessed people themselves, rather than by other community members.

## Perspectives for Overcoming the Obsession

The ideal approach to managing the obsession with raison d’être and maladaptive approaches is unestablished. People who are obsessed with raison d’être may share common psychological or psychiatric characteristics with patients with obsessive-compulsive disorders. In both conditions, the true causes may lie in the deeper unconscious mind hidden behind the manifested compulsive behaviors (Stein, 2002). Conceivable fundamental psychodynamic backgrounds in those obsessed with raison d’être include a fear of losing the meaning of their existence in the community. This fear for meaninglessness and social invisibility is inherent in almost all humans, but several predisposing factors seem to exist in some of the people suffered from problems related to raison d’être, such as chronic illnesses (Pinquart et al., 2009; Sherman and Simonton, 2012) or psychiatric conditions (Steger et al., 2008). Past memories of oppression or being abandoned by someone close and the educational environment in youth may also contribute to the development of such obsessions (Cath et al., 2008; Grisham et al., 2008). As described in the previous sections, the first step to overcome the obsession would be to be self-aware of the presence of such unconscious fears in their minds. If obsessed people can successfully notice that they are obsessed with raison d’être, the next step will be trying to unconditionally accept the existence of their and other people’s lives, regardless of the underpinning with raison d’être. These steps are difficult, as the history of mankind has demonstrated; the limited amount of resources in the world (e.g., food, land, fuel, money, spouse, or social position) would inevitably require restless competition or conflict with others for survival. The struggle for survival or species preservation would be a more fundamental and universal emotion than raison d’être. Many people who are obsessed with raison d’être may believe that raison d’être and struggle for survival are largely the same, but maladaptive pursuit for raison d’être may even oppose the chance for survival. In fact, survival or species preservation can be realized without raison d’être. In the face of losing or an absence of raison d’être, trying not to be desperate to regain it and just living without it for a while may provide relief to people who are obsessed with raison d’être and performing maladaptive behaviors.

## Data Availability Statement

The original contributions presented in the study are included in the article/supplementary material, further inquiries can be directed to the corresponding author.

## Author Contributions

TA concepted and drafted the manuscript.

## Conflict of Interest

The author declares that the research was conducted in the absence of any commercial or financial relationships that could be construed as a potential conflict of interest.

## Publisher’s Note

All claims expressed in this article are solely those of the authors and do not necessarily represent those of their affiliated organizations, or those of the publisher, the editors and the reviewers. Any product that may be evaluated in this article, or claim that may be made by its manufacturer, is not guaranteed or endorsed by the publisher.
